# Letter to the editor regarding European Headache Federation guideline on the use of monoclonal antibodies acting on the calcitonin gene related peptide or its receptor for migraine prevention

**DOI:** 10.1186/s10194-019-0975-2

**Published:** 2019-03-01

**Authors:** Russell Nichols, Vladimir Skljarevski, Grazia Dell’Agnello, Hans-Peter Hundemer, Sheena K. Aurora

**Affiliations:** Eli Lilly and Company, and/or one of its subsidiaries, Indianapolis, IN USA

To the Editor,

We were excited to see the online publication of the European Headache Federation guideline on the use of monoclonal antibodies acting on the calcitonin gene related peptide or its receptor for migraine prevention [1]. These guidelines provide timely information for clinicians, payers, and ultimately patients in the preventive treatment of migraine and including all available and pertinent information is crucial.

While reviewing the guidelines, we noticed that EVOLVE-2, one of the galcanezumab phase 3 studies in episodic migraine, was completely omitted. Because of this, all data from this study were not considered and associated levels of certainty were downgraded throughout the publication. The guidelines reference that there was a study called EVOLVE-2, however; the reference associated with EVOLVE-2 by Skljarevski et al. [2] is a phase 2 dose finding study and the data points in Table 1 correspond to that study. Skljarevski was also the primary author for the EVOLVE-2 publication in 2018 [3] and we believe this may have led to the error. Both EVOLVE-1 and EVOLVE-2 should be accounted for in the guidelines.

EVOLVE-1 and EVOLVE-2 were identically designed phase 3 studies and 26% of the patients in EVOLVE-2 were from Europe. Because there were similar/consistent endpoint results between the two, we believe that multiple sections in the guidelines need to be updated. Of note, the “certainty of the evidence (GRADE)” levels in multiple tables appear to have been downgraded since only 1 phase 3 study in episodic migraine for galcanezumab was noted. We believe that all levels of evidence and associated data for galcanezumab in episodic migraine within the article should be reviewed and updated based on the inclusion of EVOLVE-2 as a phase 3 study.

We acknowledge and thank the consensus panel and all involved in the creation of the guidelines for their countless hours and hard work. However, with this error and omission, we believe that the guidelines need to be updated to ensure an accurate and fair representation of the data.
